# Internet-based guided self-help for glioma patients with depressive symptoms: design of a randomized controlled trial

**DOI:** 10.1186/1471-2377-14-81

**Published:** 2014-04-10

**Authors:** Florien W Boele, Irma M Verdonck-de Leeuw, Pim Cuijpers, Jaap C Reijneveld, Jan J Heimans, Martin Klein

**Affiliations:** 1 Department of Medical Psychology, VU University Medical Center, Van der Boechorststraat 7, 1081 Amsterdam, BT, the Netherlands; 2 Department of Otolaryngology – Head & Neck Surgery, VU University Medical Center, Amsterdam, the Netherlands; 3 Clinical Psychology, VU University, Amsterdam, the Netherlands; 4 Department of Neurology, VU University Medical Center, P.O. Box 7057, 1007 Amsterdam, MB, the Netherlands

**Keywords:** Glioma, Brain tumor, Depression, Quality of life, Internet-based treatment, Informal caregiver

## Abstract

**Background:**

Among glioma patients, depression is estimated to be more prevalent than in both the general population and the cancer patient population. This can have negative consequences for both patients and their primary informal caregivers (e.g., a spouse, family member or close friend). At present, there is no evidence from randomized controlled trials for the effectiveness of psychological treatment for depression in glioma patients. Furthermore, the possibility of delivering mental health care through the internet has not yet been explored in this population. Therefore, a randomized controlled trial is warranted to evaluate the effects of an internet-based, guided self-help intervention for depressive symptoms in glioma patients.

**Methods/design:**

The intervention is based on problem-solving therapy. An existing 5-week course is adapted for use by adult glioma patients with mild to moderate depressive symptoms (Center for Epidemiology Studies Depression Scale score ≥12). Sample size calculations yield 126 glioma patients to be included, who are randomly assigned to either the intervention group or a waiting list control group. In addition, we aim to include 63 patients with haematological cancer in a non-central nervous system malignancy control group. Assessments take place at baseline, after 6 and 12 weeks, and after 6 and 12 months. Primary outcome measure is the change in depressive symptoms. Secondary outcome measures include health-related quality of life, fatigue, costs and patient satisfaction. In addition, all patients are asked to assign a primary informal caregiver, who does not participate in the intervention but who is asked to complete similar assessments. Their mood, health-related quality of life and fatigue is evaluated as well.

**Discussion:**

This is the first study to evaluate the effects of problem-solving therapy delivered through the internet as treatment for depressive symptoms in glioma patients. If proven effective, this treatment will contribute to the mental health care of glioma patients in clinical practice.

**Trial registration:**

Netherlands Trial Register NTR3223

## Background

Patients with gliomas, primary brain tumors originating from glial tissue, are not only faced with the diagnosis of a life-threatening disease, but also with various neurological symptoms [1,2]. Glioma patients often suffer from headaches [3-5], cognitive deficits, paresis, visual-perceptual deficits, sensory loss, and seizures [1]. In addition, depression is common in glioma patients. During the first 8 months following the diagnosis, 15 to 20% of glioma patients become clinically depressed [6,7]. Longitudinal studies show that the prevalence of depression among these patients keeps increasing up to one year after surgery [8]. Surprisingly, glioma patients not only seem to be at increased risk compared with the general population (12-month prevalence 6.6%), but also compared with other cancer patient populations [9,10]. This may affect not only patients, but also their direct environment, including their spouses, family members, and close friends. In fact, glioma patients’ neuropsychiatric status, including depressive symptoms, was found to influence the presence of depressive symptoms in significant others [11]. These mental health issues can contribute significantly to a decrease in significant others’ health-related quality of life (HRQOL) [12,13].

The precise cause of depression in glioma patients remains unclear. A number of mechanisms, including tumor location, elevated intracranial pressure, and biochemical changes may contribute to the development of depression [14]. Evidently, the patient’s emotional reactions to the diagnosis can contribute. Patients can experience shock and disbelief, dysphoria, despair, anger and anxiety, and intrusive thoughts about their diagnosis [15]. These issues may cause an adjustment disorder to the situation, which if persistent, can become a major depressive episode [16]. However, evidence for this theory is inconclusive as awareness of the prognosis is not always associated with mood disorders [17]. Moreover, these mechanisms occur in other cancer patient populations as well, which only emphasizes the unexpectedly high prevalence of depression among glioma patients, specifically.

While the etiology of this problem is not fully known, it is clear that the above mentioned contributing factors may impede the diagnosis of depression in glioma patients [18]. For example, the mood problems can be interpreted by treating physicians as ‘understandable’ considering the circumstances, and this may complicate communication about these symptoms [19]. The depression is then less likely to be recognized, which can lead to an underdiagnosis, and subsequently to an undertreatment, of depression [20]. This process can have serious negative consequences for glioma patients as in this population, depression has been associated with increased morbidity and even with poorer survival [21,22]. Moreover, depression is the most important independent predictor of HRQOL in patients with brain tumors [23].

If this potentially treatable condition is recognized, the treatment usually consists of the combination of antidepressants and intensive psychological treatment, such as cognitive behavioral therapy (CBT) [24,25]. Both pharmacological and psychological treatment can encounter problems in glioma patients specifically. Glioma patients often take multiple medications concurrently, which increases the risk for drug interactions and may lead to a reluctance to try antidepressants. For example, one study shows that only 60% of patients in whom the treating physician recognized depression, actually received antidepressants [26], and it is unclear if the remaining 40% of depressed patients received an alternative treatment such as psychotherapy. Treatment with psychotherapy can encounter problems as well, as psychotherapy usually requires good cognitive functioning in order for a patient to benefit most while approximately 80% of brain tumor patients experience cognitive deficits to some degree [1]. Therefore, while treatment for depression has been shown effective in both the general population [27,28] and in cancer patients [29], it remains to be seen if either antidepressants or psychotherapy are equally effective in glioma patients.

Problem-solving therapy (PST), a form of CBT, may prove helpful in alleviating depressive symptoms especially in this patient population, as it is a brief and practical approach. Depression is linked with stressful life events, and when depressed, patients may be less able to actively cope with these stress inducing factors. In PST, it is assumed that depressive symptoms are caused by everyday problems that can be resolved with problem-solving techniques. Resolution of problems then leads to a reduction in depressive symptoms. By teaching more adequate coping strategies, and aiding in the acceptance of problems that cannot be solved [30], PST can prove effective [31,32]. Indeed, it has been suggested as the preferred treatment in depressive patients with somatic disease [33].

During their disease trajectory, glioma patients frequently have to visit the hospital. In a subset of patients who suffer from neurological sequelae that affect their physical functioning and mobility (e.g. paresis, paralysis, epilepsy, fatigue), face-to-face treatment for depression may lead to additional burden. Alternative ways of delivering PST, such as through the internet, may therefore become more appealing. This may especially be the case in the Netherlands, where in 2013, approximately 95% of all households has internet access [34]. Internet-based psychological interventions, including PST, have already been found to be equally effective as face-to-face treatment [27,35]. These internet-based programs make use of self-help, where patients work through a standardized psychological treatment independently, sometimes guided by a coach. This way, the interventions are thought to pose low thresholds for participation, as it is more anonymous, easily accessible as patients can work on the programs at a time of their choosing, and cost-effective as only minimal involvement of health-care professionals is necessary. As of yet, there is no scientific evidence from randomized controlled trials available for the effectiveness of psychotherapy, whether internet-based or face-to-face, in glioma patients [36]. Therefore, we presently present the design of a randomized, controlled trial aimed at alleviating depressive symptoms in glioma patients using an internet-based guided self-help course. A secondary aim is to evaluate the effect of the intervention on HRQOL of both patients and their significant others.

## Methods/design

### Design

This study is a randomized controlled trial evaluating the effects of an internet-based guided self-help course for depressive symptoms in glioma patients. We compare a group of patients who receive the intervention with a three month waiting list control group and a non-central nervous system malignancy control group. The intervention, which takes approximately 5 weeks to complete, is aimed at patients with mild to moderate depressive symptoms.

**Figure 1:**
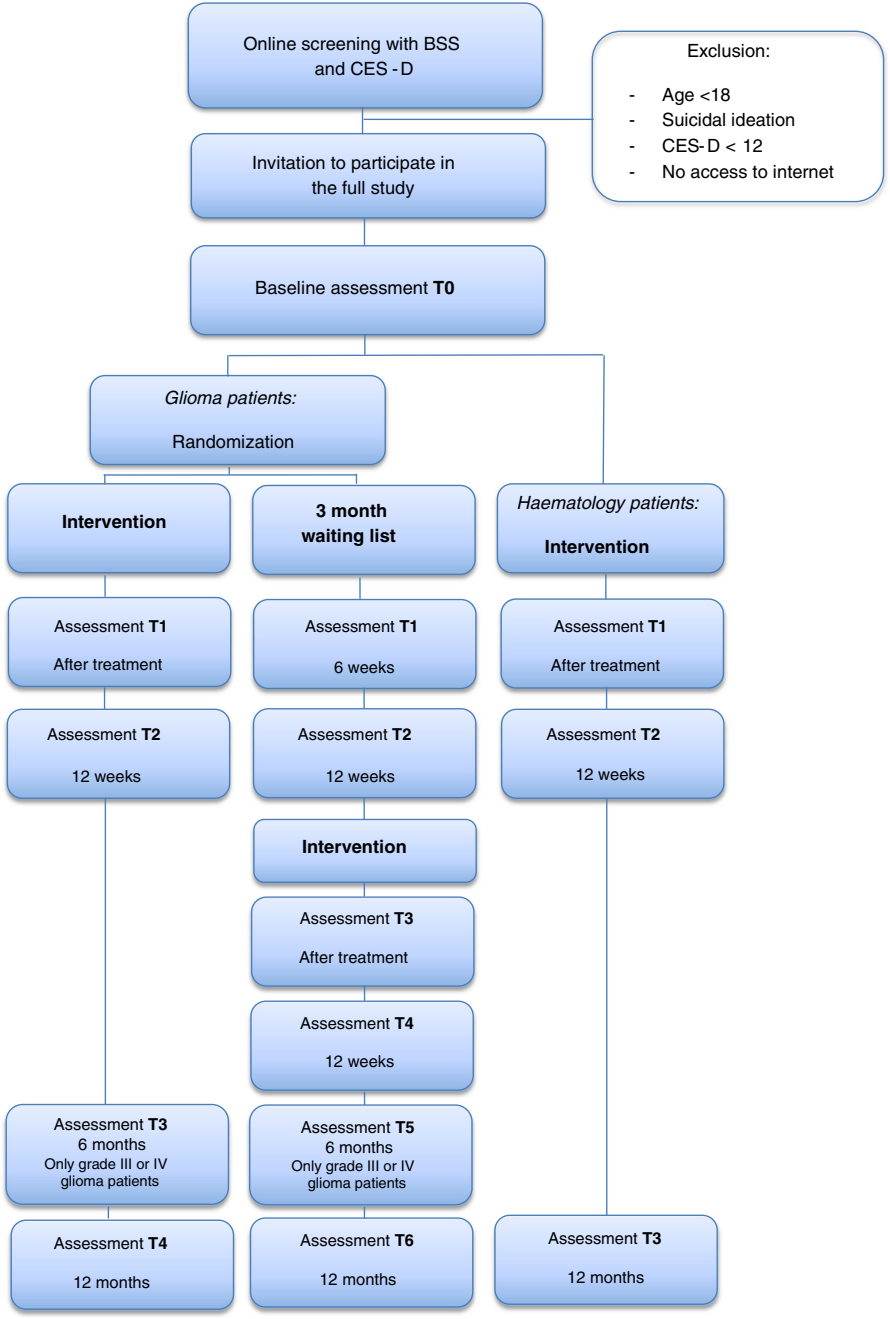
Participant flow.

Assessments include self-reported outcomes completed online. The assessments are scheduled at baseline (T0), after completion of the online course (approximately 6 weeks after baseline; T1), 12 weeks after baseline (T2), and 12 months after baseline, see Figure 1. Those in the waiting list control group receive the same assessments, but are also assessed after completion of the online course (T3; approximately 6 weeks after T2), and 12 weeks thereafter (T4). Furthermore, patients with a high-grade malignancy undergo an additional assessment at 6 months after baseline (T3 or T5), as is also depicted in Figure 1. This study is performed in accordance with the Declaration of Helsinki.

This study is approved by the institutional review board of the VU University Medical Center (IRB00002991). Due to the internet-based nature of the intervention and the assessments, this approval is deemed sufficient for nation-wide recruitment.

### Study population

Adult (>18 years of age) WHO grade II, III or IV glioma patients with mild to moderate depressive symptoms (Center for Epidemiological Studies Depression Scale [37] (CES-D) score ≥12) are invited to participate. After screening, patients scoring above 16, the usual cut-off score for depression on the CES-D, subsequently receive the full study information. Those scoring between 12 and 16 are informed of their relatively low score and are given the option to receive the full study information or not.

For the non-central nervous system oncology control group, adult (>18 years of age) patients with non-Hodgkin lymphoma (NHL), chronic lymphatic leukaemia (CLL), multiple myeloma (MM), or a myelodysplastic syndrome (MDS) who have mild to moderate depressive symptoms (CES-D score ≥12) are invited to participate. Here, too, those patients scoring between 12 and 16 on the CES-D at the time of screening are informed of their relatively low score and they are asked if they want to receive full information of the study or not.

Although not a strict requirement for participation, all patients are additionally asked to invite an informal caregiver to participate in the study. This refers to a significant other who provides the majority of mental and physical support to the patient. These informal caregivers do not participate in the intervention but are asked to complete the same assessments at the same time points concerning their own mood, HRQOL, fatigue, etc.

Potential participants are excluded if they have no access to the internet and/or no email address, if they have insufficient proficiency of the Dutch language, and if they express suicidal intent. Suicidal intent is screened for with the Beck Scale for Suicide Ideation (BSS) [38]. If patients have a score higher than 0 on the BSS, a board certified psychologist (MK) conducts an interview through telephone to assess the severity of symptoms. If a patient is excluded due to suicidal intent, the general practitioner is always contacted to assure proper referral to health care professionals.

### Recruitment and inclusion procedure

Patients are recruited through advertisements and news items on websites frequently visited by glioma patients and haematological patients. Patient associations are asked for help in spreading study information through their websites, newsletters and meetings. Treating physicians and nurse practitioners throughout the Netherlands collaborate in this study by providing information brochures to their patients. These professionals are contacted through the intermediary of the Dutch Society for Neuro-Oncology and the Dutch Neurology Association, as well as through the authors’ personal networks.

The advertisements, news items and information brochures contain a link to a website with an online screening procedure. If interested, patients complete this online questionnaire that contains questions on basic demographic information (age, gender, level of education), contact information, and the BSS and CES-D. On this website, it is explained that by completing the questionnaire, patients give permission to contact their general practitioner if the researchers deem this necessary. Furthermore, they are made aware that the personal information they provide will only be used if they later decide to take part in the study and sign informed consent forms.

**Figure 2:**
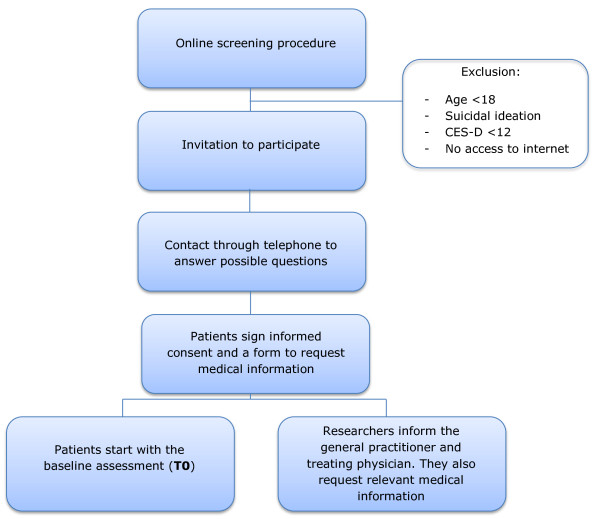
Recruitment procedure.

Eligible patients receive an information letter. Within a week the study coordinator (FWB) contacts the eligible patients by telephone to answer possible questions. Subsequently, they are asked to sign the informed consent form they received with the information letter. They are also asked to sign a form enabling the researchers to request information on the patient’s disease and treatment at their treating hospital. Figure 2 illustrates this recruitment procedure. After informed consent is obtained, both the general practitioner and the treating physician are informed about the patient’s intent to participate in the trial.

### Randomization

Glioma patients are randomly assigned to either the intervention group or the waiting list control group after completion of the baseline assessment (T0). An adaptive simple randomization technique is employed to minimize the chance of imbalanced group sizes.

### Intervention

The internet-based guided self-help intervention is based on the principles of problem solving therapy. The original intervention, ‘Everything under control’ (“*Alles onder controle*”) has been shown to have significant positive effects on depression, anxiety and stress/burnout in a randomized controlled trial with 215 adults from the general population [39]. With small changes, this online intervention is adapted for use by glioma patients and patients with haematological malignancies. Modifications concern additional information about the specific diseases and their treatment, and the psychological impact on everyday life. Examples of participants’ assignments are made disease-specific.

The intervention consists of 5 modules with text and exercises. Patients are asked to complete one module a week and spend a minimum of two hours a week on their exercises. During the intervention, patients describe what they feel is important in their lives, make a list of their problems and concerns, and divide these into three categories: 1) unimportant problems (problems that are not related to what is important in their life), 2) important and solvable, and 3) important but unsolvable. For each of these problems the patient makes a plan on how to cope with this, guided by methods explained in the modules. The participants receive feedback on the exercises from a personal coach within three working days after completion of the assignment. The coach is not a therapist, but only supports the patient in working through the intervention. Participants can always contact their coach for additional support through the website. The coaching is provided by one of the researchers (FWB), by trained and supervised students in the final phase of a Master’s program in Psychology or by specialists from Prezens. In collaboration with the VU University medical center, Prezens provides psychological care and support.

The glioma patients in the waiting list control group are offered the same intervention after completion of the 12 weeks follow-up (T2).

### Outcome measures

Self-report measures of depressive symptoms, HRQOL, fatigue, costs and patient satisfaction are presented online in a fixed order. Patients are allowed to return to any of the measures for review or changes during an assessment.

### Primary outcome measure

The primary outcome measure is the change in depressive symptoms as measured with the CES-D [37]. This questionnaire is designed to measure depressive symptoms in the general population (i.e., persons older than 18, without psychiatric disease). The 20-item scale measures the major components of depressive symptomatology, including depressive mood, feelings of guilt and worthlessness, psychomotor retardation, loss of appetite, and sleep disturbance. Participants are asked to indicate if they feel a particular item is applicable to their situation of the past week, on a 4-point scale. Scores range between 0 and 60, with higher scores indicating more depressive symptoms. In the general population, the usual cut-off score for depression is ≥16. Within cancer patients, the CES-D has yielded good psychometric properties, with good construct validity, good internal consistency and proper test-retest reliability [40].

### Secondary outcome measures

#### Suicidal intent (BSS)

The Beck Scale for Suicide Ideation (BSS) [38] is a 21-item self-report instrument for detecting and measuring the current intensity of the patients’ specific attitudes, behaviors, and plans to commit suicide during the past week. The first 19 items consist of three options graded according to the intensity of the suicidal intent and rated on a 3-point scale ranging from 0 to 2. These ratings are then summed to yield a total score, which ranges from 0 to 38. The last two items assess the number of previous suicide attempts and the seriousness of the intent to die associated with the last attempt. In this study, only the first 19 items are administered. If the patient reports any active or passive desire to commit suicide on the first 5 screening items, then the additional 14 items are administered as well.

#### Health-related quality of life (SF-36 and EQ-5D)

HRQOL is assessed by means of the Short-Form Health Survey (SF-36) [41]. The SF-36 is composed of 36 items, organized into 8 multi-item scales assessing: (1) physical functioning; (2) role functioning-physical; (3) role functioning-emotional; (4) pain; (5) vitality; (6) social functioning; (7) mental health; and (8) general health perceptions. Scores range from 0-100. Furthermore, two higher-order summary scores can be computed – one for physical health (Physical Component Summary) and one for mental health (Mental Component Summary). On these scales, scores have a mean of 50 and a standard deviation of 10 based on data from the general population.

In addition, the EuroQol (EQ-5D) [42] is administered. This is a standardized, non-disease specific instrument assessing HRQOL. With 5 items scored on a 3-point Likert type scale, the EQ-5D measures mobility, self-care, usual activities, pain/discomfort and anxiety/depression.

#### Brain tumor-specific HRQOL (EORTC BN20)

For glioma patients, a supplementary questionnaire module is employed to assess additional health problems associated specifically with brain tumors and their treatment. The EORTC Brain Cancer Module (BN20) [43] is organized into multi-item subscales assessing future uncertainty, visual disorders, motor dysfunctions, and communication deficits. The remaining 7 items assess other disease symptoms and side-effects of treatment found to be prevalent among patients with brain tumors, including headaches, seizures, drowsiness, hair loss, itching, weakness in the legs, and lack of bladder control. The scores range from 0-100, with higher scores indicating more symptoms.

#### Fatigue (CIS)

Fatigue and fatigue related symptoms are measured with the Checklist Individual Strength (CIS) [44]. The CIS is a multidimensional fatigue scale; it measures fatigue severity (8 items), concentration problems (5 items), reduced motivation (4 items), and reduced activity (3 items). Each item is scored on a seven-point Likert scale. Total scores of every subscale are obtained by adding the individual items, with high scores indicating a high level of fatigue, a high level of concentration problems, low motivation, and a low level of activity. Based on data from patients with chronic fatigue syndrome, patients with a score of >35 on the fatigue severity subscale are considered to be severely fatigued.

#### Cognitive functioning

Patient’s self-reported cognitive functioning is rated by the scale developed for use in the Medical Outcomes Study [45]. This 6-item scale assesses day-to-day problems in cognitive functioning including difficulty with reasoning and problem solving, slowed reaction time, forgetfulness, and problems with concentration (range 1-6).

#### Costs (TIC-P)

Costs in terms of health care utilization and production loss are assessed with the Trimbos/iMTA questionnaire for Costs associated with Psychiatric Illness (TIC-P) [46]. This questionnaire consists of two parts; part 1 covers the direct costs of care utilization of participants (15 items), and part 2 is used to determine indirect costs that result from production loss associated with the psychiatric symptoms. Here, the Short Form Health and Labor Questionnaire [47] is incorporated. This questionnaire contains three modules that assess the absence from paid employment, production loss without absence from paid employment, and impediments to paid or unpaid employment.

#### Patient satisfaction

During the post-intervention assessment (T1 or T3), the patients’ experience with the online course is evaluated. A short study-specific questionnaire evaluating the usability, readability, course content, and self-perceived usefulness of both the online course and the feedback provided by the coach is presented along the other questionnaires. Room for remarks is provided as well.

### Statistical analyses

Appropriate parametric and non-parametric statistical tests will be employed to examine differences between the groups in terms of all relevant demographic and clinical variables at baseline. Missing observations at follow-up will be imputed using the multiple imputation procedure [48]. Following the CONSORT guidelines, the intention-to-treat principle will be applied. All randomized participants will be included in the analyses, regardless of how many treatment modules or sessions they complete. Within-group and between-group differences (e.g., patients with or without epileptic seizures, pain, neurological deficits, or self-reported cognitive deficits) in dependent variable scores will be analyzed using both univariate and multivariate statistical techniques. For between-group statistical comparisons, sociodemographic variables (age, gender, and education) will be used as covariates, where necessary. The effects of the interventions will be tested by means of Helmert contrasts. Relative improvements in depressive symptoms (CES-D score) in the experimental group compared with pre-treatment assessment as well as both control groups will be calculated using Cohen’s d. If the primary outcome measure is non-normally distributed, the test and the 95% confidence intervals will be based on robust standard errors and/or on non-parametric bootstrap techniques. This will help to correctly ascertain the relative effectiveness of the treatment over the control conditions.

### Sample size

The effect of the intervention on symptoms of depression is the primary outcome measure and this is used as starting point for the sample size calculations. Based on previous experience with this intervention in adults, we expect a Cohen’s d of 0.50. Assuming an alpha of 0.05 and a statistical power (1-beta) of 0.80, we need 50 patients in each condition. Allowing for a dropout percentage of 25% once included, we aim to recruit 126 glioma patients (63 per group) and 63 patients with haematological malignancies in total.

## Discussion

In this paper, we describe the study design of a randomized controlled trial aimed at evaluating the effects of an internet-based, guided self-help intervention for depressive symptoms in glioma patients. This study is innovative in two ways: 1) as of yet, there is no evidence from RCTs for the effectiveness of psychotherapy in glioma patients with depressive symptoms; and 2) to our knowledge, providing psychosocial care through the internet has not been explored in the depressed glioma patient population until now.

We expect the external validity of this study to be high, as we employ only few exclusion criteria. Any adult WHO grade II, III or IV glioma patient at any disease stage with mild depressive symptoms is invited to participate in the trial to try and relief these symptoms. Furthermore, as the intervention is administered through the internet and patient inclusion is organized nation-wide, we should be able to reach a large group of potential participants. Using the internet also evidently has its downsides. For certain patients, such as those unaccustomed to using the computer in their everyday lives or those with more severe cognitive impairment, using the internet may prove more difficult and could possibly result in non-participation or dropout of the study. We aim to construct the website in a very straightforward and easy-to-use way, in order to minimize this possibly negative effect. In addition, both interventions using the internet and self-help interventions are prone to high dropout rates [49], suggesting that this problem could occur in our trial as well. Therefore we present the online questionnaires separately from the intervention website, and email reminders are sent for both the assessments and the course assignments separately. Moreover, if participants do not respond to these reminders, we try and contact them through telephone instead. This should strengthen the relationship between the participants and the researchers substantially, thereby possibly improving patient participation throughout the trial [50].

Our frequent follow-up assessments, up until 12 months after the guided self-help course, enable evaluation of both short- and long-term effects of the intervention. However, frequent evaluation of mental well-being through self-reported questionnaires may also lead to patients’ increased awareness of their depressive symptoms. To minimize this possible effect we keep track of the patients’ suicidal ideation and depressive symptoms throughout follow-up and we will contact both the participant and their general practitioner in case symptoms worsen significantly.

Due to its nationwide design, our study could raise awareness of depressive symptoms among both physicians and patients. Whereas this is favorable for patients, it may lead to a risk of contamination in this randomized controlled trial, as more psychological help may be offered outside of the intervention offered in our study. However, the TIC-P assessment records all contact with health-care professionals, so with adequate power we will be able to correct for this in the analyses.

In conclusion, we currently present the design of our study aimed at improving symptoms of depression in glioma patients using a brief internet-based guided self-help intervention based on the principles of PST. This can provide methodological clarity and can aid and encourage further research efforts to improve mood and HRQOL in glioma patients. Our randomized controlled trial is expected to contribute substantially to the existing literature as there is a hiatus in studies evaluating the effectiveness of different treatments for depression, as well as interventions delivered by means of the internet in this patient population. Furthermore, implementation in clinical practice should be relatively easy to accomplish if our intervention proves to be effective, as it only requires minimal involvement of health care professionals. Currently, this trial is in the recruitment phase.

## Abbreviations

BN20: EORTC brain cancer module; BSS: Beck scale for suicide ideation; CES-D: Center for epidemiological studies depression scale; CBT: Cognitive behavioral therapy; CIS: Checklist individual strength; CLL: Chronic lymphatic leukemia; EQ-5D: EuroQol; HRQOL: Health-related quality of life; MDS: Myelodysplastic syndrome; MM: Multiple myeloma; NHL: Non-Hodgkin lymphoma; PST: Problem-solving therapy; SF-36: -Form health survey; TIC-P: Trimbos/iMTA questionnaire for costs associated with psychiatric illness.

## Competing interests

The authors declare that they have no competing interests.

## Authors’ contributions

FWB participated in the design of the study, is the study coordinator, and drafted the manuscript. IMVdL, JCR and MK conceived of the study, participated in its design and coordination and helped to draft the manuscript. PC and JJH participated in the study design, and helped to draft the manuscript. All authors read and approved the final manuscript.

## Pre-publication history

The pre-publication history for this paper can be accessed here:

http://www.biomedcentral.com/1471-2377/14/81/prepub
